# Effect of elevated carbon dioxide on shoal familiarity and metabolism in a coral reef fish

**DOI:** 10.1093/conphys/cow052

**Published:** 2016-11-09

**Authors:** Lauren E. Nadler, Shaun S. Killen, Mark I. McCormick, Sue-Ann Watson, Philip L. Munday

**Affiliations:** 1 College of Science and Engineering, James Cook University, Townsville, Queensland 4811, Australia; 2 ARC Centre of Excellence for Coral Reef Studies, James Cook University, Townsville, Queensland 4811, Australia; 3 Institute of Biodiversity, Animal Health and Comparative Medicine, University of Glasgow, Glasgow G12 8QQ, UK

**Keywords:** Calming effect, carbon dioxide, familiarity, respiratory physiology, shoaling, social recognition

## Abstract

Atmospheric CO_2_ is expected to more than double by the end of the century. The resulting changes in ocean chemistry will affect the behaviour, sensory systems and physiology of a range of fish species. Although a number of past studies have examined effects of CO_2_ in gregarious fishes, most have assessed individuals in social isolation, which can alter individual behaviour and metabolism in social species. Within social groups, a learned familiarity can develop following a prolonged period of interaction between individuals, with fishes preferentially associating with familiar conspecifics because of benefits such as improved social learning and greater foraging opportunities. However, social recognition occurs through detection of shoal-mate cues; hence, it may be disrupted by near-future CO_2_ conditions. In the present study, we examined the influence of elevated CO_2_ on shoal familiarity and the metabolic benefits of group living in the gregarious damselfish species the blue-green puller (*Chromis viridis*). Shoals were acclimated to one of three nominal CO_2_ treatments: control (450 µatm), mid-CO_2_ (750 µatm) or high-CO_2_ (1000 µatm). After a 4–7 day acclimation period, familiarity was examined using a choice test, in which individuals were given the choice to associate with familiar shoal-mates or unfamiliar conspecifics. In control conditions, individuals preferentially associated with familiar shoal-mates. However, this association was lost in both elevated-CO_2_ treatments. Elevated CO_2_ did not impact the calming effect of shoaling on metabolism, as measured using an intermittent-flow respirometry methodology for social species following a 17–20 day acclimation period to CO_2_ treatment. In all CO_2_ treatments, individuals exhibited a significantly lower metabolic rate when measured in a shoal vs. alone, highlighting the complexity of shoal dynamics and the processes that influence the benefits of shoaling.

## Introduction

Atmospheric CO_2_ has risen to >400 ppm (Dlugokencky and Tans, 2016) because of human activity, higher than any time in the last 800 000 years (Masson-Delmotte *et al*., 2013). The partial pressure of CO_2_ (pCO_2_) in the world's oceans is rising at approximately the same rate as in the atmosphere (Doney *et al*., 2009; Le Quéré *et al*., 2013). If current anthropogenic CO_2_ emissions continue unabated, average CO_2_ levels in the atmosphere and surface ocean will more than double from present-day levels by the year 2100 (Fabry *et al*., 2008; Collins *et al*., 2013). Furthermore, new models indicate that seasonal cycles in ocean pCO_2_ will be amplified in the future, meaning that marine organisms will experience extended periods of ocean pCO_2_ in excess of 1000 µatm by the end of this century (McNeil and Sasse, 2016). Rising CO_2_ levels are predicted to affect a range of behavioural (Briffa *et al*., 2012; Nagelkerken and Munday, 2016) and physiological processes (Pörtner *et al*., 2004; Heuer and Grosell, 2014) in marine organisms, with potentially far-reaching effects on marine ecosystems (Wittmann and Pörtner, 2013).

Higher environmental CO_2_ levels can be a problem for marine organisms because they act to acidify the blood and tissues and thus affect pH-dependent physiological processes (Pörtner *et al*., 2004). Fish defend against acidosis in a high-CO_2_ environment by actively regulating acid–base-relevant ions in their blood and tissues (Heuer and Grosell, 2014). Consequently, they are able to maintain a pH suitable for cellular processes, even at very high ambient CO_2_ levels (Ishimatsu *et al*., 2008; Esbaugh *et al*., 2012, 2016). However, this acid–base regulation leads to changes in extracellular ion concentrations that may interfere with the function of neurotransmitter receptors (Nilsson *et al*., 2012). These neurological changes can lead to altered behaviour and impaired sensory systems. Behavioural effects of exposure to high CO_2_ include reduced learning ability (Jutfelt *et al*., 2013; Chivers *et al*., 2014), altered activity levels (Munday *et al*., 2010; Ferrari *et al*., 2011a), higher anxiety (Hamilton *et al*., 2014), disrupted behavioural lateralization (Domenici *et al*., 2011; Jutfelt *et al*., 2013) and reduced predator avoidance behaviour (Dixson *et al*., 2010; Munday *et al*., 2010; Ferrari *et al*., 2011b). Behavioural responses to visual (Ferrari *et al*., 2012b; Chung *et al*., 2014), olfactory (Munday *et al*., 2009b) and auditory cues (Simpson *et al*., 2011; Rossi *et al*., 2016) are all affected, although one study found that visual cues were less affected than olfactory preferences at projected near-future CO_2_ levels (Lönnstedt *et al*., 2013). Some behavioural traits appear to be unaffected by elevated CO_2_, particularly foraging behaviour and swimming kinematics (Munday *et al*., 2009c; Nowicki *et al*., 2012; Maneja *et al*., 2015). In addition, some species, such as the Atlantic cod (*Gadus morhua*), exhibit tolerance to elevated CO_2_ in terms of behavioural effects (Jutfelt and Hedgärde, 2013, 2015). Even among closely related coral reef fish, there is substantial variability among species in the degree of behavioural effects in response to elevated CO_2_ (Ferrari *et al*., 2011a).

The effects of elevated pCO_2_ and decreased pH on other physiological characteristics are unclear. Theoretically, the energetic cost of increased regulatory mechanisms (such as acid–base balance regulation) should manifest in higher overall energetic needs (Ishimatsu *et al*., 2008). However, studies measuring standard metabolic rate (SMR; the metabolic rate of a resting, fasting and non-stressed individual; a measure of basic energetic needs) of fishes under elevated pCO_2_ have found highly variable results (reviewed by Heuer and Grosell, 2014; Lefevre, 2016), reporting increases (Munday *et al*., 2009a; Enzor *et al*., 2013), decreases (Rummer *et al*., 2013) and no effects of pCO_2_ on SMR (Deigweiher *et al*., 2008; Melzner *et al*., 2009; Strobel *et al*., 2012; Couturier *et al*., 2013), suggesting that the effects may be species or context specific. However, another important consideration is that, although many studies have examined the effect of pCO_2_ on the metabolic rate of gregarious fish species (Munday *et al*., 2009a; Miller *et al*., 2012; Rummer *et al*., 2013), all have measured metabolic rate in solitary individuals, which can have effects on the measured metabolic rate because of the stress of isolation (Nadler *et al*., 2016). Therefore, how social context may modulate the effect of pCO_2_ on metabolic traits, such as SMR, remains unknown. Recent work found that the immediate social environment can have a significant impact on metabolic rate, with individuals tested in the presence of shoal-mate cues exhibiting a significantly lower minimal measured metabolic rate than individuals tested in social isolation (Nadler *et al*., 2016). One factor that is likely to contribute to this calming effect is a reduced need for individual vigilance, because animal groups exhibit improved threat detection by having ‘many eyes’ to scan for predators (Roberts, 1996; Ward *et al*., 2011). Individuals accustomed to a social environment may also exhibit reduced stress when allowed to associate with conspecifics (Hennessy *et al*., 2009). The importance of these benefits could increase in the presence of environmental stressors, such as rising pCO_2_, because having a reduced metabolic rate in shoaling conditions could aid in coping with the projected rise in energy demand associated with changing environmental conditions.

Group living is widespread among fish species and carries benefits for individuals with respect to predator avoidance, foraging opportunities and energy use (Shaw, 1978; Krause and Ruxton, 2002). A learned familiarity can be attained following a prolonged period of interaction between social individuals (reviewed by Ward and Hart, 2003), increasing the probability of reciprocal cooperation between members of an animal group (Granroth-Wilding and Magurran, 2013). This greater cooperation can have benefits for a range of fitness-enhancing processes and characteristics, including foraging, social learning, body condition and survival (Seppä *et al*., 2001; Swaney *et al*., 2001; Atton *et al*., 2014). As a result, fish prefer to shoal with familiar conspecifics (e.g. Magurran *et al*., 1994; Griffiths and Magurran, 1997; Bhat and Magurran, 2006; Edenbrow and Croft, 2012), with individual identification achieved primarily through olfactory stimuli (Partridge and Pitcher, 1980; Brown and Smith, 1994; Ward *et al*., 2002). As elevated pCO_2_ is known to impact behavioural traits and sensory abilities necessary for social recognition, the ability to recognize familiar shoal-mates may be compromised in future environmental conditions.

Elevated pCO_2_ may affect the calming effect and the ability of fish to recognize conspecifics owing to its effects on fish behaviour, sensory abilities or physiology. In the present study, we examined the effect of elevated pCO_2_ on familiarity and the calming effect in the blue-green puller, *Chromis viridis* (Cuvier, 1830), a common species of shoaling damselfish. Shoals were acclimated to one of the following three CO_2_ treatments: control (450 µatm), mid-CO_2_ (750 µatm) or high-CO_2_ (1000 µatm). Our first aim was to determine whether elevated pCO_2_ modulated familiarity, using a choice test in which individuals were given the choice to associate with familiar shoal-mates or unfamiliar conspecifics. Our second aim was to explore whether the calming effect was altered by environmental pCO_2_, using an intermittent-flow respirometry methodology for social species. We hypothesized that familiarity would be disrupted by elevated pCO_2_. Given the known benefits of familiarity to shoaling fish (Seppä *et al*., 2001; Swaney *et al*., 2001; Atton *et al*., 2014), we also predicted that the calming effect on the minimal measured metabolic rate would be reduced if familiarity was disrupted at elevated pCO_2_.

## Materials and methods

### Fish collection and maintenance

Experiments were conducted at the Lizard Island Research Station in the northern Great Barrier Reef (14°40′08″S; 145°27′34″E). Shoals of *C. viridis* (standard length, 3.22 ± 0.03 cm; body mass, 1.29 ± 0.04 g; mean values ± SEM) were collected from reefs in the lagoon adjacent to the Lizard Island Research Station using hand nets and barrier nets. *Chromis viridis* is an abundant, live coral-associated shoaling species found on coral reefs throughout the Indo-Pacific region in groups ranging in size from a few to hundreds of individuals (Randall *et al*., 1997). Fish were placed into groups composed of eight individuals and housed in replicate 30 litre aquaria in a flow-through seawater system. All experimental shoals were held together for a minimum of 15 days to ensure that they exhibited a uniform degree of familiarity (Ward *et al*., 2003). Fish were fed to satiation twice daily with INVE Aquaculture pellets and newly hatched *Artemia* sp.

### Carbon dioxide treatments and administration

Shoals were acclimated to one of the following three CO_2_ treatments: 450 µatm (ambient control), 750 µatm or 1000 µatm (4–7 days for behaviour experiments and 17–20 days for physiology experiments; seawater chemistry summarized in Table 1). These elevated-CO_2_ treatments were chosen based on the range of CO_2_ levels projected for the year 2100 (Collins *et al*., 2013; McNeil and Sasse, 2016). The CO_2_ administration methodologies followed standard procedures for ocean acidification research (Gattuso *et al*., 2010). The only deviation from this prescribed methodology was the use of single header tanks for each CO_2_ treatment (Cornwall and Hurd, 2015), as space limitations in the field prevented us from having multiple header tanks for each CO_2_ treatment. Seawater was pumped directly from the ocean into each 60 litre header tank. Elevated-CO_2_ seawater treatments were achieved by dosing CO_2_ to a set pH, using a pump placed into each header tank through which CO_2_ was diffused. This pump aided in rapid dissolution of CO_2_ and vigorous stirring of water in the header tank. A pH controller (Aqua Medic, Germany) attached to each CO_2_ treatment header tank maintained pH at the desired level. In control header tanks, air was diffused through sump pumps. Equilibrated seawater was then pumped at a rate of ~700 ml/min to each of the replicate 30 litre experimental tanks. For each of these replicate tanks, seawater pH_NBS_ (pH measured on the NBS scale; Mettler Toledo SevenGo Pro) and temperature (Comark C22) were recorded daily. Seawater CO_2_ was confirmed with *in situ* CO_2_ measurements, using a portable CO_2_ equilibrator and non-dispersive infrared (NDIR) sensor (Vaisala GMP343; Hari *et al*., 2008; Munday *et al*., 2014b). For experiment 1, *in situ* CO_2_ measurements were conducted once weekly in the control and 1000 µatm treatments to confirm CO_2_ levels based on pH measurements. During experiment 2, these measurements were conducted on each treatment at least three times weekly, during which CO_2_ measures were recorded. These measurements are detailed in Table 1 and confirm our calculated pCO_2_. Salinity was measured by an automated float in the Lizard Island lagoon (Bainbridge, 2015). Water samples were taken twice weekly and analysed for total alkalinity by Gran titration (888 Titrando, Metrohm, Switzerland) to within 1% of certified reference material (Professor A. Dickson, Scripps Institution of Oceanography). Average pCO_2_ was calculated with the program CO2SYS, from measured pH_NBS_, temperature, salinity and total alkalinity, using constants from Mehrbach *et al*. (1973) refitted by Dickson and Millero (1987) and Dickson (1990) for KHSO_4_.

**Table 1:** Summary of seawater chemistry parameters in control and elevated-carbon dioxide treatments for experiments 1 and 2

Treatment	Experiment no.	Temperature (°C)	Salinity (psu)	pH_NBS_	Total alkalinity [μmol (kg seawater)^−1^]	pCO_2_ (calculated, μatm)	pCO_2_ (*in situ*, μatm)
Control CO_2_	1	28.8 (±0.2)	35.5 (±0.01)	8.15 (±0.010)	2284 (±1)	442 (±9)	465 (±13)
	2	28.9 (±0.3)	35.0 (±0.03)	8.13 (±0.002)	2309 (±8)	461 (±2)	449 (±11)
Mid CO_2_	1	29.1 (±0.2)	35.5 (±0.01)	7.96 (±0.001)	2285 (±12)	734 (±5)	–
	2	29.0 (±0.3)	35.0 (±0.03)	7.96 (±0.001)	2296 (±8)	753 (±2)	766 (±11)
High CO_2_	1	28.8 (±0.2)	35.5 (±0.01)	7.86 (±0.001)	2296 (±2)	963 (±7)	981 (±17)
	2	28.8 (±0.3)	35.0 (±0.03)	7.87 (±0.001)	2297 (±11)	952 (±3)	983 (±15)

The estimated partial pressure of CO_2_ (Estimated pCO_2_) was calculated in the program CO2SYS using the other measured parameters. *In situ* pCO_2_ was measured using a portable CO_2_ equilibrator with non-dispersive infrared (NDIR) sensor. Seawater pH was measured on the NBS (National Bureau of Standards) scale (pHNBS). Error is SEM.

### Experiment 1: effect of elevated CO_2_ on familiarity

**Figure 1:**
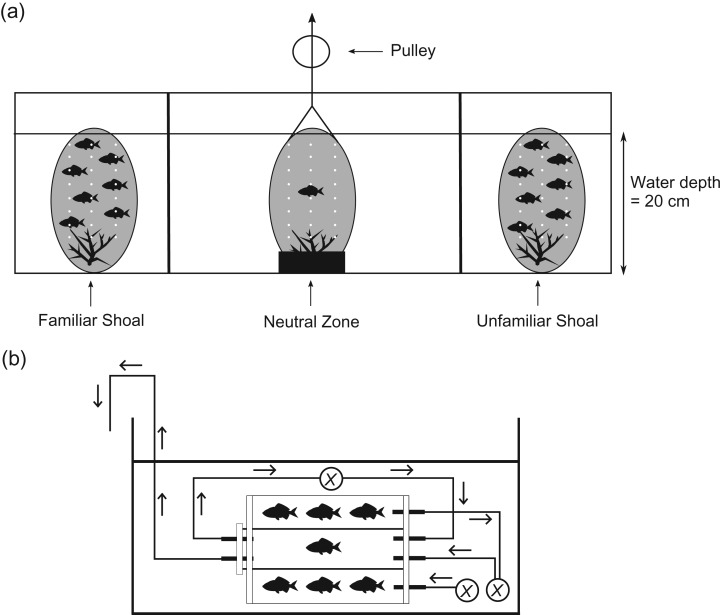
Schematic diagrams of the two experimental set-ups. (**a**) Choice test tank used in experiment 1 (90 cm length × 30 cm width × 30 cm height). The dark ovals on either end of the tank represent the shoal holding containers, and the dark oval in the centre of the tank illustrates the container used for the focal fish during the pre-trial acclimation period. White dots represent the porosity of the containers (each container contained 50 5 mm holes). (**b**) Side view of the respirometry chamber. The experimental set-up was composed of an inner respirometry chamber (length, 13.5 cm; inner diameter, 3.24 cm; volume of chamber and associated gas-impermeable tubing, 100 ml) and an outer shoal-mate holding chamber (length, 12.0 cm; inner diameter, 11.4 cm; volume of chamber, 1.10 litres). Arrows indicate the direction of water flow through tubing. Each *X* indicates a water pump used for mixing the inner chamber and flushing both chambers. The outer shoal-mate holding chamber was flushed with its own pump. The outflow port for this outer chamber was connected to the flush pump for the inner respirometry chamber, to provide olfactory cues of shoal-mates to the focal individual. In order to ensure proper mixing in the inner respirometry chamber, a pump ran continuously in a closed loop. Deoxygenated water in the inner chamber was discarded during on phases of the flush pump. All focal individuals were tested in both an alone-testing treatment and a shoal-testing treatment (with six shoal-mates).

Nine experimental *C. viridis* shoals, each composed of eight fish, were acclimated to each CO_2_ treatment for a period of 4–7 days before experimentation. This time period is sufficient for elevated CO_2_ to induce behavioural changes in reef fishes, and previous studies indicate that longer acclimation periods do not change results (Munday *et al*., 2013a, 2014a; Welch *et al*., 2014). Two individuals per group were chosen randomly for testing for shoal association preferences (*n* = 18 individuals per treatment). These individuals were distinguished from each other and their shoal-mates using unique visible implant elastomer (VIE) tags (Hoey and McCormick, 2006). The VIE tags were administered 24–48 h before placement in the CO_2_ treatment. Shoaling preference was established using a choice test, using methodology adapted from Griffiths and Magurran (1997). An elongate testing tank (Fig. 1a) was filled to a depth of 20 cm with seawater at the same CO_2_ level as the relevant treatment. Two 1 litre plastic containers (height, 24 cm × diameter, 10 cm) were placed at each end of the tank, 6 cm from the side-wall. The plastic containers were transparent and made porous to olfactory cues by holes drilled around the circumference (50 5 mm holes per container). Shoals composed of 7 fish of either the familiar or an unfamiliar group were placed in these bottles. The location of the familiar shoal (right or left bottle) was randomized. The shoal used as unfamiliar was also randomized, to ensure that each shoal within a treatment was used as the unfamiliar shoal a uniform number of times and that a different unfamiliar shoal was used when testing each of the two focal fish from a shoal. The focal fish was placed in a clear, porous container in the centre of the tank. This container sat over a small coral shelter, and the bottom 3 cm of the container was opaque to allow the fish to take shelter. All fish were left to acclimate in this container for 15 min, which was a sufficient time period for all fish to calm down after handling. The container surrounding the focal fish was then lifted using a pulley system so that the focal fish would not be disturbed by visual cues of the observer. Trials lasted 15 min and were video-recorded (Canon Powershot D10). Pilot trials were conducted with food colouring to estimate the degree of olfactory cue mixing throughout the choice test tank during the 30 min trial (including both the 15 min acclimation period and the 15 min testing period). While there was olfactory mixing in the neutral zone of the experimental tank (Fig. 1a), no mixing occurred in the shoal association zones within this time frame.

Using QuickTime Player 7 (v 7.6.6), videos were analysed for the following factors: (i) the proportion of time spent shoaling with each group; (ii) initial shoal choice following removal of the barrier; and (iii) total shoal visits (a proxy for activity, which indicates the number of times that the focal fish traversed the experimental tank). Individuals were said to be shoaling when they were swimming within two body lengths of the shoal (Pitcher and Parrish, 1993). To ensure that focal fish were making an informed choice (e.g. had experienced the sensory cues of both stimulus shoals), they had to visit both shoal preference zones within a trial or they were retested the next day (occurred with 22% of focal fish across CO_2_ treatment groups). Different unfamiliar shoals were used when retesting to prevent learning of unfamiliar conspecifics. Activity was recorded so that we could confirm that any effect of CO_2_ on shoal association preferences was not attributable to changes in activity levels between treatments.

### Experiment 2: effect of elevated CO_2_ on the calming effect

Ten experimental shoals were acclimated to each CO_2_ treatment for a period of 17–20 days. This longer acclimation period was used for this experiment because studies show that metabolism requires a longer period of time to adjust to elevated CO_2_ treatments (Enzor *et al*., 2013). One individual per group was chosen randomly for testing (*n* = 10 individuals per treatment) and was identified using VIE tags (Hoey and McCormick, 2006). The VIE tags were administered 24–48 h before placement in the CO_2_ treatment.

The calming effect was measured using a previously described intermittent-flow respirometry methodology for social species (Nadler *et al*., 2016; Fig. 1b). Respirometry is a technique in which oxygen uptake rates are measured as a proxy for aerobic metabolism (Steffensen, 1989; Nelson, 2016). Each respirometry chamber was composed of two cylindrical glass tubes: an inner tube (length, 13.5 cm; inner diameter, 3.24 cm; total volume of chamber and associated gas-impermeable tubing, 100 ml) and an outer tube (length, 12.0 cm; inner diameter, 11.4 cm; total volume of chamber minus volume occupied by inner chamber, 1.10 litres). The outer chamber was affixed to the exterior of the inner chamber and was used to provide visual and olfactory cues of shoal-mates to the focal individual. This larger chamber was aerated with a continuously running flush pump. To provide olfactory cues of shoal-mates to the focal individual, the water leaving the outflow port was attached to the inflow vent for the inner chamber's flush pump. The inner chamber was connected to a recirculating pump (to mix water in the respirometer) and a flushing pump that flushed the chamber with oxygen-saturated water for 3 min between each 9 min measurement period. The water used to flush the chamber between measurement periods was maintained at the same pH and pCO_2_ as the focal fishes’ treatment. Chambers were immersed in separate, temperature-controlled water baths (29 ± 0.5°C). Temperature was maintained through a combination of air conditioning and controlling ambient water flow to the water bath. The metabolic rate of each focal fish was recorded in an alone-testing treatment (no shoal-mates in the outer chamber) and a group-testing treatment (six shoal-mates in the outer chamber). The order of testing trials (testing of the alone or group treatment first) was randomized. All focal fish were given 48 h between testing trials.

Dissolved oxygen concentration in the inner, focal chamber was measured every 2 s and logged using a Fire-Sting fibre-optic oxygen meter (Pyroscience, Germany), connected to a computer. The oxygen-sensing optode was mounted in the recirculation loop in a flow-through cell, to ensure that flow was sufficient for a fast response time of the sensor (Svendsen *et al*., 2016). Focal fish were fasted for 24–26 h before experimentation to ensure that they were in a post-absorptive state and were left undisturbed in the respirometers for 17–19 h overnight, as *C. viridis* is quiescent at night. A dim light remained on through the night in the laboratory to simulate moonlight, allowing the focal fish to see their shoal-mates in group testing trials. Activity was recorded during daylight hours using a webcam (H264 Webcam software) and was measured by counting the number of 180° turns for 10 min/h of testing (from which turns/min was calculated). Activity was recorded to ensure that any measured effects of CO_2_ on oxygen uptake were not attributable to changes in activity between CO_2_ treatments. Slopes (*s*) were calculated from plots of oxygen concentration vs. time using linear least-squares regression (LabChart v6) and converted to the rate of oxygen uptake (${\dot{M}}_{O_{2}}$; in milligrams of O_2_ per hour). For all trials, background respiration was measured in empty chambers for three measurement periods both before and after trials. Microbial respiration was then subtracted from all fish respiration measurements, assuming a linear increase in microbial respiration over time (Rodgers *et al*., 2016).

Once focal individuals had completed both the alone- and group-testing trials, maximum metabolic rate (MMR) was measured in separate trials, so that each individual's aerobic scope (AS) could be calculated. The AS is an individual's aerobic metabolic capacity, which indicates the available energy that an individual has for all aerobic processes beyond basic maintenance (Farrell, 2016). The MMR was measured using the chase protocol, in which individuals are exercised to exhaustion through manual chasing (Roche *et al*., 2013). Although this method may not always provide the highest estimates of MMR (Roche *et al*., 2013), it is an accepted and repeatable method for determining a relative value for MMR between individuals. Fish were considered exhausted when they no longer responded to chasing by burst swimming. Fish were then air exposed for 30 s to ensure that they had depleted all endogenous oxygen stores. Individuals were then transferred to their respective respirometry chambers, and oxygen uptake was measured for 8–10 min (this time frame was used to ensure that oxygen saturation in the water remained >80% air saturation; Hughes, 1973). This method elicits anaerobic exercise in individuals, and maximal rates of oxygen uptake were measured during subsequent recovery. The MMR was measured for all fish in an alone-testing treatment. These oxygen uptake slopes were measured at 3 min intervals, with the greatest oxygen uptake during this period taken as MMR.

Three measures of metabolic rate were analysed. First, the minimal measured metabolic rate in fish exposed to each treatment (MR_min_) was estimated using the protocol typically employed to measure SMR in the literature. This was accomplished by taking MR_min_ as the lowest 10th percentile of all ${\dot{M}}_{O_{2}}$ measurements (Killen, 2014; Chabot *et al*., 2016), and comparisons were drawn between individuals tested alone and with a group. Second, routine metabolic rate (RMR; the metabolic rate of an undisturbed animal, including costs of random activity) was calculated as the mean ${\dot{M}}_{O_{2}}$ excluding the first 5 h in the respirometer, and differences between fish tested alone (RMR_alone_) and fish tested in groups (RMR_group_) were assessed (Killen *et al*., 2011). These 5 h were excluded from RMR calculations because pilot trials determined that ${\dot{M}}_{O_{2}}$ in *C. viridis* takes an average of 5 h to stabilize (SS. Killen, LE. Nadler, MI. McCormick, unpublished data). Third, individuals’ response to stress was also determined by using the first slope (FS) of each alone- and group-testing trial, following transfer to the respirometer. The stress response was calculated in the context of AS (AS = MMR − MR_min_), in order to determine the proportion of AS that fish were using in response to stress (the stressor in this case being handling stress during transfer to the respirometer). The initial stress response (ISR) was therefore calculated using the following equation:

$$\mathrm{ISR}\;=\;(\mathrm{FS}\;-\;{\mathrm{MR}}_{\min})/\mathrm{AS}$$

The ${\dot{M}}_{O_{2}}$ is commonly used as an indicator of stress and reaction to threats, such as predation, because of the previously established link between oxygen uptake and stress hormones, including cortisol and epinephrine, with oxygen uptake increasing as the concentration of stress hormones rises (e.g. Brown *et al*., 1982; Morgan and Iwama, 1996). In the present study, the stressor was the handling stress induced during transfer to the respirometer and any stress of being in isolation.

### Statistical analysis

Statistical analysis was conducted in the R Statistical Environment (v. 3.2.4) using the packages ‘nlme’, ‘multcomp’, ‘lme4’ and ‘car’ (Bates and Maechler, 2009; R Development Core Team, 2015; Pinheiro *et al*. 2016). For experiment 1, three separate models were conducted, to determine the preference for the familiar shoal within each treatment (as measured by the proportion of time spent with the familiar shoal). As the null hypothesis is 0.5 (which would indicate no preference for either shoal), the deviation from 0.5 for each observation was used as the response variable, and differences in deviation from 0 were assessed in general linear mixed-effects models (LMMs), with shoal number as a random effect (so that each individual was nested within their experimental shoal). Differences in activity (total shoal visits) between treatments were tested using an LMM, with CO_2_ treatment as a fixed effect and shoal number as a random effect. To ensure that all assumptions were met, homogeneity of variance and normality were assessed through visual inspection of the residual and quantile–quantile (Q-Q) plots, respectively. No transformations were necessary to meet assumptions. Initial shoal choice was tested using an LMM with a binomial distribution, with CO_2_ treatment as a fixed effect and shoal number as a random effect.

For experiment 2, differences in the MR_min_, ISR and activity were analysed using an LMM, with CO_2_ treatment and testing treatment (alone or group) as fixed effects, body mass as a covariate (to account for differences in size between individuals), and individual as a random effect. In statistical analysis, whole-animal metabolic rate values were used. In figures, metabolic rate measures were mass corrected by plotting the residual values for each measure from the relationship between the logarithm of body mass (in grams) and the logarithm of metabolic rate (in milligrams of O_2_ per hour). Each residual was added to the fitted value for mass = 1.29 g, the mean mass of all fish used in the study. Significant differences in CO_2_ treatments (which had three levels) discovered using LMM were investigated further using Tukey's multiple comparisons *post hoc* tests. Differences in MMR and AS with CO_2_ treatment were examined using a generalized linear model (GLM), with body mass as a covariate. For these models, assumptions of homogeneity and normality were again checked through visual inspection of residual and Q-Q plots. No transformations were necessary to conform to these assumptions.

## Results

### Experiment 1: effect of elevated CO_2_ on social recognition

**Figure 2:**
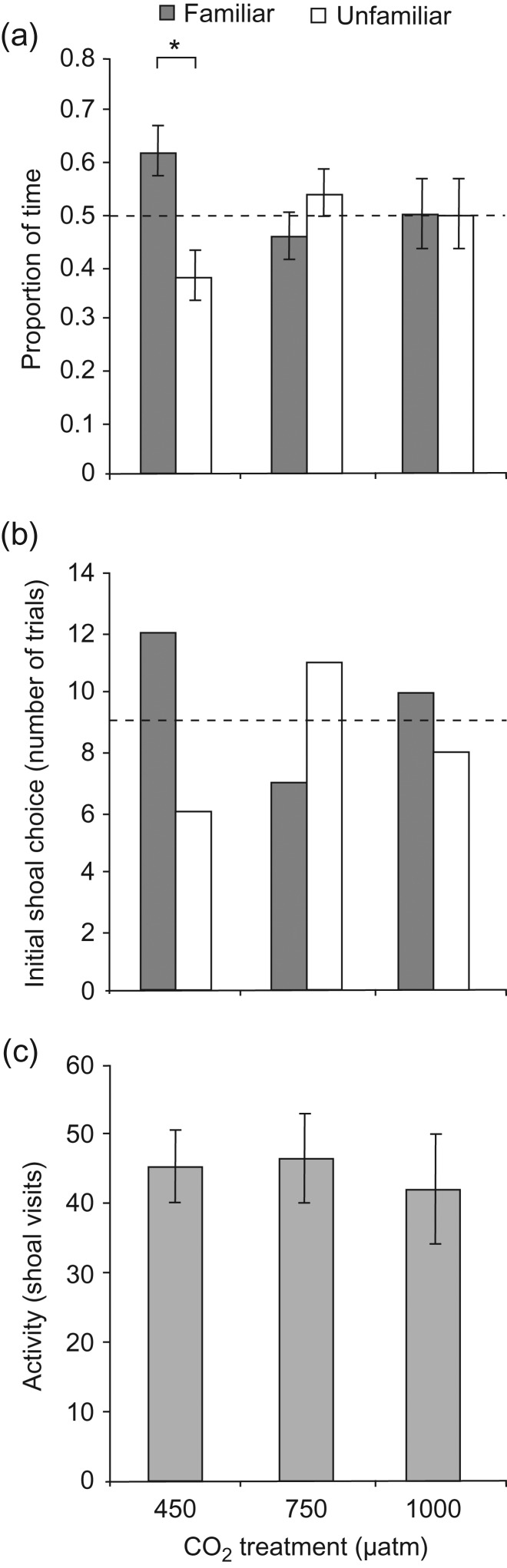
Effect of CO_2_ on shoal preference and activity. (**a**) Proportion of time spent with each shoal (familiar and unfamiliar). (**b**) Initial shoal choice after removal of the barrier (in number of trials). (**c**) Mean activity per trial (number of shoal visits). Error bars are SEM, and *n* = 18 for all treatments. Asterisks indicate statistical significance (*P < 0.05).

Individuals exhibited a significant preference for the familiar shoal in control conditions, but this preference was lost in both elevated-CO_2_ treatments (Fig. 2a; 450 µatm, *F*_1,10_ = 6.10, *P* = 0.033; 750 µatm, *F*_1,10_ = 0.660, *P* = 0.438; and 1000 µatm, *F*_1,10_ = 0.001, *P* = 0.991). Trends in initial shoal choice matched those found for shoal preference, but the effect of CO_2_ treatment on initial shoal choice was not statistically significant (Fig. 2b; χ^2^ = 0.8103, *P* = 0.368). Total shoal visits were not significantly different between the CO_2_ treatments (Fig. 2c; *F*_2,25_ = 0.1138, *P* = 0.893), with individuals exhibiting an overall mean of 44.7 shoal visits per trial (range, 2–133 shoal visits per trial).

### Experiment 2: effect of elevated CO_2_ on the calming effect

**Figure 3:**
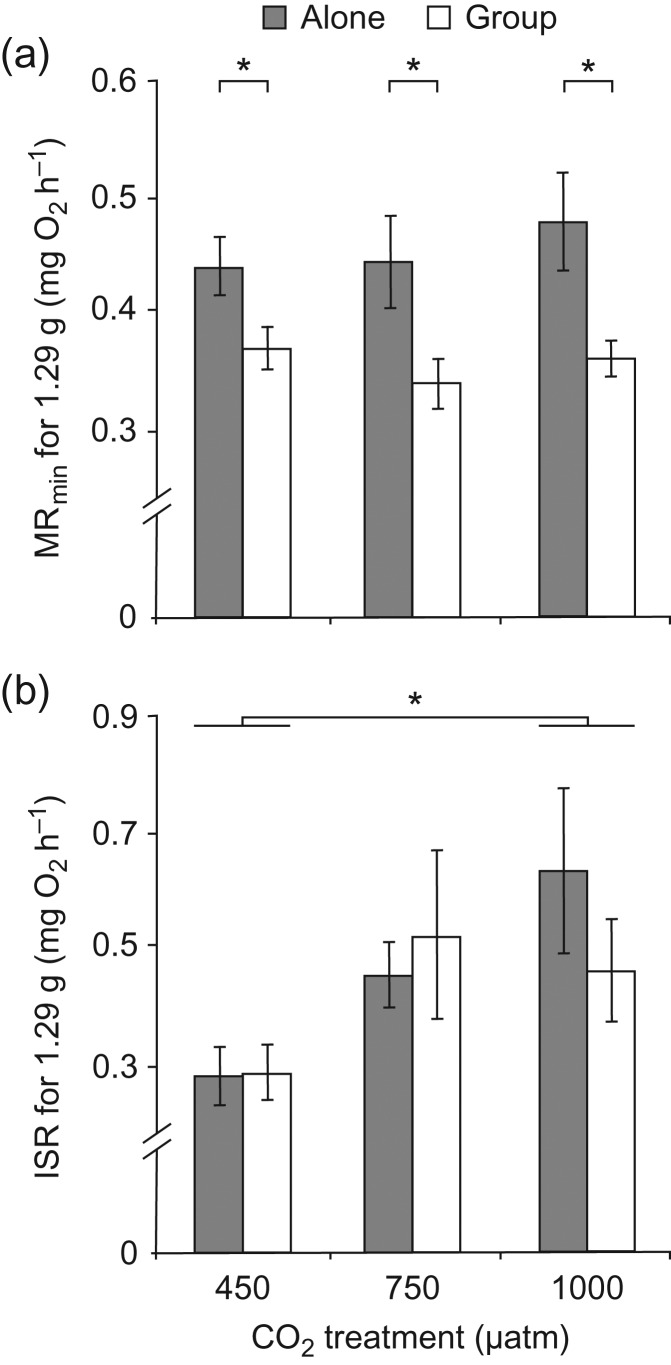
Effect of CO_2_ and testing treatment on the minimal metabolic rate (MR_min_; in milligrams of O_2_ per hour; **a**) and initial stress response (ISR; in milligrams of O_2_ per hour; **b**). In these panels, metabolic rate measures were mass corrected by using residuals of the relationship between logarithm of the body mass and logarithm of whole-animal metabolic rate added to the fitted value for mass = 1.29 g, the mean mass of all fish used in the study. Error bars are SEM, and *n* = 10 for all treatments. Asterisks indicate statistical significance (*P < 0.05). Statistical analysis was conducted on whole-animal metabolic rates, with body mass as a covariate.

The MR_min_ tested in a group was significantly lower than MR_min_ tested alone (Fig. 3a; *F*_1,26_ = 29.01, *P* < 0.001), regardless of CO_2_ treatment (Fig. 3a; *F*_2,27_ = 0.37, *P* = 0.698), with 26 out of 30 fish tested exhibiting an average reduction in MR_min_ of 22.8% (the remaining four fish exhibited an average increase in MR_min_ of 10.5% when tested in a group; these four fish were included in all statistical analyses). The interaction between testing and CO_2_ treatment was not significant (*F*_2,26_ = 0.71, *P* = 0.501); however, the magnitude of the calming effect was higher in both elevated-CO_2_ treatments than it was in control conditions (450 µatm, 13.9 ± 5.6%; 750 µatm, 21.4 ± 4.2%; and 1000 µatm, 19.8 ± 7.3%; Fig. 3a). Elevated-CO_2_ treatments produced a trend towards higher ISR (Fig. 3b; *F*_2,27_ = 2.94, *P* = 0.069), with differences attributable to a significant increase in ISR from the control to the high-CO_2_ treatment (Tukey's test: 450 vs. 1000 µatm, *P* = 0.028; for all other comparisons, *P* > 0.05). The ISR was not affected by testing treatment (*F*_1,26_ = 0.27, *P* = 0.606).

**Figure 4:**
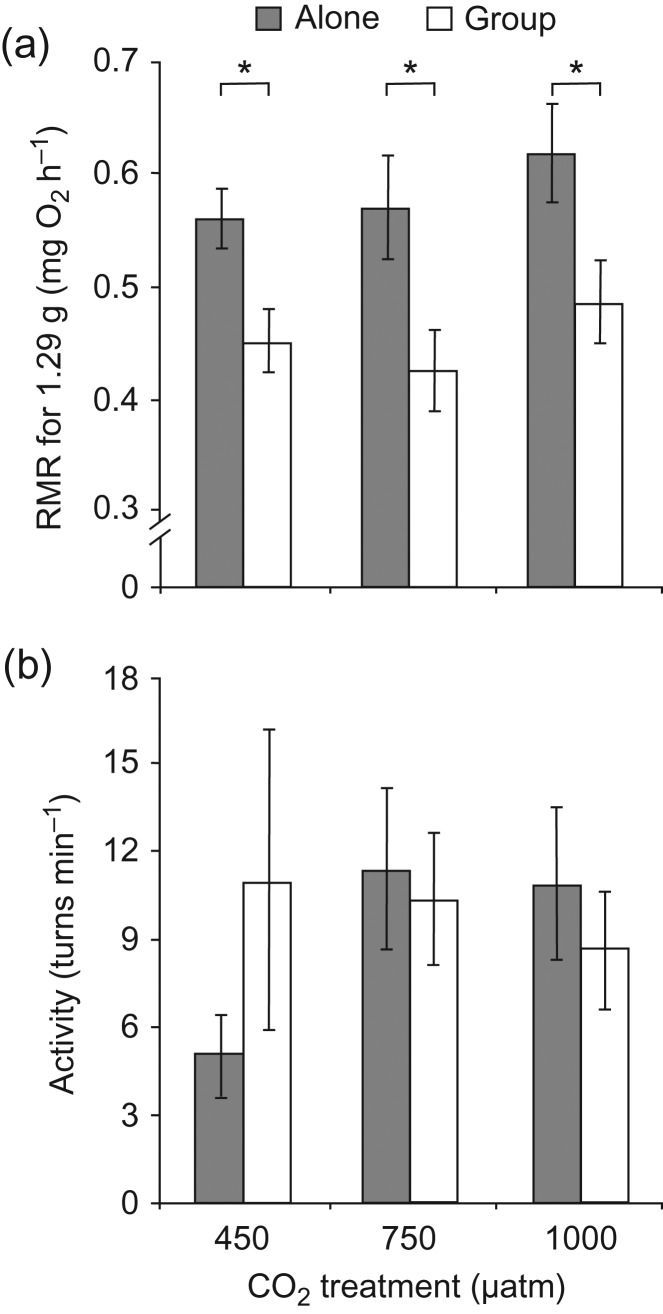
Effect of CO_2_ and testing treatment on the routine metabolic rate (RMR; in milligrams of O_2_ per hour; **a**) and activity (number of 180**°** turns/min; **b**). In these panels, metabolic rate measures were mass corrected by using residuals of the relationship between the logarithm of the body mass and logarithm of whole-animal metabolic rate added to the fitted value for mass = 1.29 g, the mean mass of all fish used in the study. Error bars are SEM, and *n* = 10 for all treatments. Asterisks indicate statistical significance (*P < 0.05). Statistical analysis was conducted on whole-animal metabolic rates, with body mass as a covariate.

**Figure 5:**
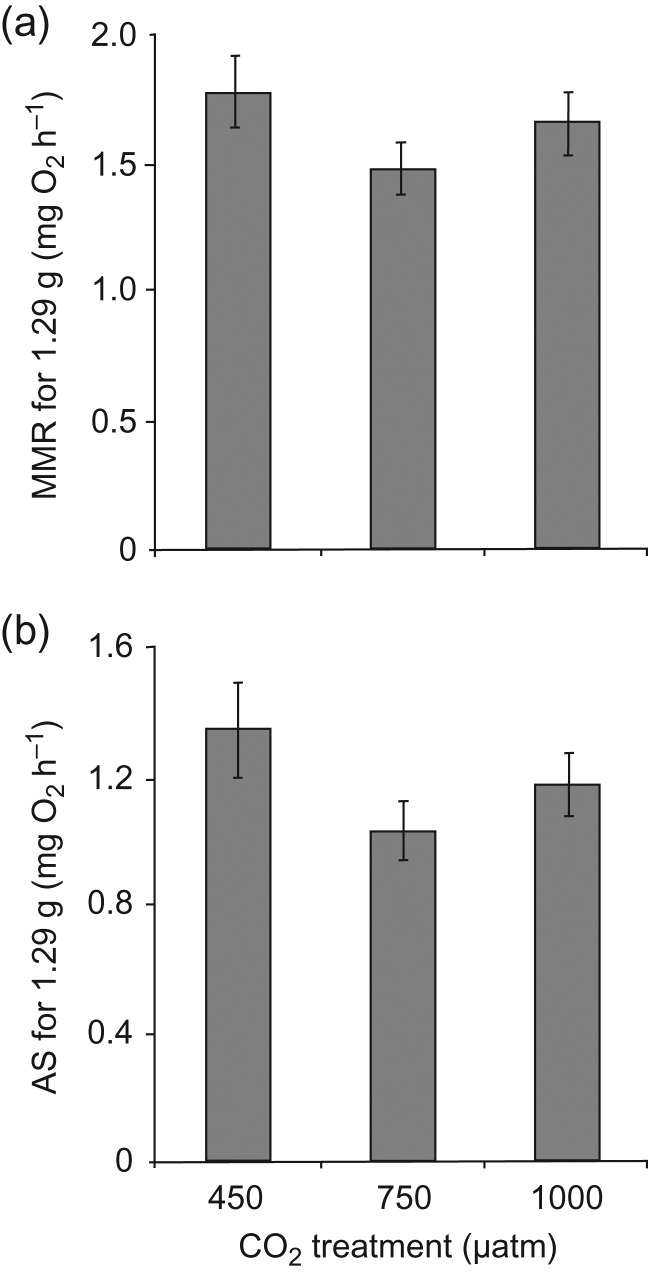
Effect of CO_2_ on maximal metabolic rate (MMR; in milligrams of O_2_ per hour; **a**) and aerobic scope (AS; in milligrams of O_2_ per hour; **b**). In these panels, metabolic rate measures were mass corrected by using residuals of the relationship between the logarithm of body mass and logarithm of whole-animal metabolic rate added to the fitted value for mass = 1.29 g, the mean mass of all fish used in the study. Error bars are SEM, and *n* = 10 for all treatments. Statistical analysis was conducted on whole-animal metabolic rates, with body mass as a covariate.

The RMR_group_ was significantly lower than RMR_alone_ (Fig. 4a; *F*_1,26_ = 33.84, *P* < 0.001). Respirometry treatment had a comparable effect on RMR in individuals from all CO_2_ treatments (*F*_2,27_ = 0.73, *P* = 0.490), and there was no significant interaction between testing and CO_2_ treatment (*F*_2,26_ = 0.43, *P* = 0.714). Neither testing (*F*_1,26_ = 0.31, *P* = 0.583) nor CO_2_ treatment (*F*_2,27_ = 0.32, *P* = 0.732) exerted a significant effect on activity (Fig. 4b), with individuals exhibiting an overall mean of 9.5 turns/min during daylight hours (range, 0.5–55.7 turns/min). The MMR (*F*_1,27_ = 1.15, *P* = 0.294) and AS (*F*_1,27_ = 1.93, *P* = 0.176) were not significantly different between CO_2_ treatments (Fig. 5).

## Discussion

Elevated CO_2_ disrupted familiarity, but not the calming effect, in *C. viridis*. As familiarity is important for a range of processes in shoaling fish (Ward and Hart, 2003), many of the benefits of group living may be altered in changing environmental conditions. However, the calming benefit of shoaling on metabolic rate was maintained in high-CO_2_ conditions, indicating that the benefits of group living on overall metabolic demand will be likely to persist under projected future pCO_2_.

The loss of familiarity with elevated CO_2_ could have occurred as a result of a number of possible mechanisms. First, social recognition may have been disrupted if fish lost the sensory abilities necessary for identifying individuals, particularly by olfactory cues (Partridge and Pitcher, 1980; Brown and Smith, 1994; Ward *et al*., 2002; Munday *et al*., 2009b). The changes in shoal-mate association found with rising CO_2_ in the present study are consistent with previous work that tested for preferences between conspecifics from different reefs (home vs. foreign reef site) in the cardinalfish, *Cheilodipterus quinquelineatus* (Devine *et al*., 2012). In that study, fish lost the association for conspecifics from their home reef under elevated CO_2_, suggesting that association preferences generally may be altered. Alternatively, individuals may still be able to recognize familiar shoal-mates, but may simply have lost the preference to shoal with familiar rather than unfamiliar individuals. Many previous studies have established that shoaling fish prefer to group with familiar conspecifics (e.g. Magurran *et al*., 1994; Griffiths and Magurran, 1997; Bhat and Magurran, 2006; Edenbrow and Croft, 2012), but few have investigated what factors may cause this preference to be lost (Granroth-Wilding and Magurran, 2013). Neural circuitry is likely to contribute to the development of social behaviour and preferences in fish species (Dreosti *et al*., 2015). As neurotransmitter function may be impaired by elevated pCO_2_ conditions (Nilsson *et al*., 2012; Heuer and Grosell, 2014), this effect may account for the loss of preferential association with familiar shoal-mates. In addition, memory and learning play an integral role in familiarity, by allowing individuals to learn about their shoal-mates and remember their identity. Although it is known that learning is interrupted by elevated CO_2_ (Ferrari *et al*., 2012a; Chivers *et al*., 2014), no studies have yet examined effects on fish memory. Nevertheless, a disruption to memory could account for the loss of association preference found here in the high-CO_2_ treatments.

These mechanisms of familiarity disruption could have a number of ecological implications. If social recognition is disrupted, as a result of either a loss of sensory abilities or a loss of memory, a number of important processes may be affected. First, social learning may be impaired as individuals are unable to distinguish between informed and naïve shoal-mates (Swaney *et al*., 2001). Second, Galhardo *et al*. (2012) found that personality traits, such as exploratory behaviour and boldness, decrease in fishes in unfamiliar shoals, suggesting that disruption to social recognition could impact fishes’ personality traits. Third, defensive behaviours may become less effective, as unfamiliar shoals are slower to react to a predator threat than familiar shoals (Griffiths *et al*., 2004). Alternatively, if only the preference for the familiar shoal is lost, a range of traits related to shoaling dynamics could be impacted. First, shoal fidelity may decrease, because, without the preference for the familiar shoal, the trade-offs of staying with the familiar shoal vs. migrating to a more suitable, unfamiliar shoal may shift (Muleta and Schausberger, 2013). Second, cooperation between shoal-mates may decrease, because individuals’ perception of shoal-mates could shift from that of a collaborator to a competitor in this different social context as the reliability of reciprocal cooperation may be compromised (Granroth-Wilding and Magurran, 2013; Engelmann and Herrmann, 2016).

Given the benefits of familiarity to a range of important shoaling processes, including foraging and social learning (Seppä *et al*., 2001; Swaney *et al*., 2001; Atton *et al*., 2014), we expected the magnitude of the calming effect to suffer under elevated CO_2_. However, unlike familiarity, the calming effect was maintained, and even enhanced, under high CO_2_. This surprising result implies that familiarity and the calming effect may rely on different mechanisms. Previous studies have highlighted the central role of olfactory sensing abilities in social recognition of familiar shoal-mates (Partridge and Pitcher, 1980; Brown and Smith, 1994; Ward *et al*., 2002), which appear to be more vulnerable to the effects of elevated CO_2_ than the visual system (Lönnstedt *et al*., 2013). Therefore, unlike familiarity, the calming effect may be able to compensate for olfactory impairments using visual cues, as has previously been found for anti-predator behaviours (Lönnstedt *et al*., 2013). The importance of shoaling to energy budgets could increase in the presence of environmental stressors, as evidenced by the increasing magnitude of the calming effect with higher pCO_2_. Any reduction in metabolic demands (like those induced by shoaling) could aid in coping with the projected rise in energy demand associated with changing environmental conditions. In addition, no effect of CO_2_ was found on any of the metabolic traits measured (including MR_min_, RMR, MMR and AS). Although some studies have indicated an effect of CO_2_ on metabolism, most have not, indicating that the results presented here are consistent with many of the studies in the literature (Lefevre, 2016).

The initial physiological reaction to stress increased with high CO_2_. This result is consistent with greater incidences of anxious behaviour in fish exposed to elevated CO_2_ (Hamilton *et al*., 2014). In social species, such as *C. viridis*, this amplified stress response could stem from the mechanisms presented above for familiarity. If social recognition or memory were lost, individuals may have perceived their shoal-mates to be unfamiliar, owing to the inability to distinguish between individuals, although this effect was not evident between the alone- and group-testing treatments. Stress hormones, such as cortisol, increase when individuals are exposed to an unfamiliar shoal (Yue *et al*., 2006), which could account for the greater acute stress response that was measured with high CO_2_. Conversely, the increased metabolic stress response may have contributed to the loss of preference for familiar shoal-mates. Shoaling motivation increases with stress and predation risk (Croft *et al*., 2009; Stier *et al*., 2013); therefore, the desire to shoal may outweigh the strategic choice to shoal with familiar fish in elevated-CO_2_ conditions. No matter what the underlying mechanism is, these results indicate that shoaling may become even more important in altered environmental conditions, with the potential to be used as a behavioural compensatory mechanism (Connell and Ghedini, 2015).

Overall activity (total shoal visits and number of 180° turns) did not vary in either experiment in response to CO_2_ treatment, indicating that differences in activity cannot explain the results found. Previous studies have reported a range of findings on the effect of CO_2_ on activity. For instance, Munday *et al*. (2014a) reported an increase in the activity of reef fish species, and Regan *et al*. (2016) found a reduction in the activity of a river catfish species. In contrast, Nowicki *et al*. (2012) found no effect of elevated CO_2_ on general activity in clownfish, and Munday *et al*. (2016) measured no effect in larvae of a pelagic kingfish species. These trends imply that CO_2_ may have variable effects on activity depending on a range of traits, such as the natural mobility of the study organism, ontogenetic stage and environmental conditions.

As with all ocean acidification research, these results must be viewed in the context in which the study was conducted. This type of study must be conducted in the laboratory in order to expose fish to controlled, elevated-CO_2_ conditions. Although every effort is made to make these conditions as realistic as possible, the laboratory setting may impart unknown effects on our results. Importantly, fishes will incrementally reach projected CO_2_ conditions over a period of many decades, so there may be the potential for acclimation or adaptation over this time period (Munday *et al*., 2013b). A longer exposure period to elevated CO_2_ might lead to different effects on behaviour. Parental exposure to elevated CO_2_ does not appear to ameliorate impairments to a number of relevant behavioural traits and sensory systems (Welch *et al*., 2014), but whether adaptation could reduce the behavioural effects of high CO_2_ over longer time frames is unknown.

Future research should work to tease apart which mechanism (social recognition, preference for familiarity or memory) is more likely to be causing the effect of CO_2_ on familiarity. Familiarity is important for many aspects of shoaling dynamics (Swaney *et al*., 2001; Griffiths *et al*., 2004), so its disruption may create further carry-over effects on a range of processes. The maintenance of the calming effect in the presence of high CO_2_, however, highlights the complexity of shoal dynamics and illustrates that many processes, in addition to familiarity, influence the benefits of shoaling.
